# Opioid-Related Diagnoses and Concurrent Claims for HIV, HBV, or HCV among Medicare Beneficiaries, United States, 2015

**DOI:** 10.3390/jcm8111768

**Published:** 2019-10-24

**Authors:** Man-Huei Chang, Ramal Moonesinghe, Lyna Z. Schieber, Benedict I. Truman

**Affiliations:** 1 National Center for HIV/AIDS, Viral Hepatitis, STD, and TB Prevention, Centers for Disease Control and Prevention (CDC), Atlanta, Georgia, GA 30329, USA; 2 Office of Minority Health and Health Equity, CDC, Atlanta, Georgia, GA 30341, USA; 3 National Center for Injury Prevention and Control, CDC, Atlanta, Georgia, GA 30341, USA

**Keywords:** health status disparities, socioeconomic factors, infectious disease medicine, Medicare

## Abstract

Unsterile opioid injection increases risk for infection transmission, including HIV, hepatitis B virus (HBV), or hepatitis C virus (HCV). We assess prevalence of and risk factors associated with opioid overdose and infections with HIV, HBV, or HCV among Medicare beneficiaries with opioid-related fee-for-service claims during 2015. We conducted a cross-sectional analysis to estimate claims for opioid use and overdose and HIV, HBV, or HCV infections, using data from US Medicare fee-for-service claims. Beneficiaries with opioid-related claims had increased odds for HIV (2.3; 95% confidence interval (CI), 2.3–2.4), acute HBV (6.7; 95% CI, 6.3–7.1), chronic HBV (5.0; 95% CI, 4.7–5.4), acute HCV (9.6; 95% CI, 9.2–10.0), and chronic HCV (8.9; 95% CI, 8.7–9.1). Beneficiaries with opioid-related claims and for HIV, HBV, or HCV infection, respectively, had a 1.1–1.9-fold odds for having a claim for opioid overdose. Independent risk factors for opioid overdose and each selected infection outcome included age, sex, race/ethnicity, region, and residence in a high-vulnerability county. Having opioid-related claims and selected demographic attributes were independent, significant risk factors for having HIV, HBV, or HCV claims among US Medicare beneficiaries. These results might help guide interventions intended to reduce incidences of HIV, HCV, and HBV infections among beneficiaries with opioid-related claims.

## 1. Introduction

The US opioid crisis has increased incidence of opioid-related harms, unsterile injection-drug use (IDU), infections with hepatitis C (HCV), hepatitis B (HBV), HIV, and other outcomes [1,2]. In 2017, the Centers for Disease Control and Prevention (CDC) estimated a total of 70,237 drug overdose deaths, of which 47,600 (67.8%) involved opioids, and during 2016–2017, synthetic opioid-involved overdose death rates increased 45.2% [3].

Illicit opioid injection increases risk for infectious disease transmission, especially bloodborne infections [4]. Approximately 9% of 39,782 new diagnoses of HIV in the United States during 2016 were attributable to IDU or male-to-male sexual contact and IDU [5]. Bloodborne infections are more prevalent and often more severe among persons addicted to opioids than among the general population [6,7,8].

HCV infection is the underlying or contributing cause of more deaths than any other infection [9], and the number of new HCV infections tripled during 2010–2016 [10]. The nationwide increase in acute HCV infection is related to the country’s opioid overdose epidemic and associated increases in IDU [11,12,13]. A 133% increase in acute HCV cases during 2004–2014 was associated with increases in opioid injection [14], and an estimated 20,900 new cases of acute HBV infection occurred during 2016 [10].

National data indicate increasing opioid misuse among older adults [15]. Effects among older adults who take prescription medications [16,17,18] and vulnerable populations (e.g., persons who inject drugs) in economically disadvantaged areas [19] have been described. Opioid-related inpatient stays and emergency department visits among patients aged ≥65 years increased 34.3% and 74.2%, respectively, during 2010–2015 [16]. Recently, the Centers for Medicare and Medicaid Services (CMS) released proposed changes for the Medicare health and Part D benefit plans to reduce opioid overdose risk [20].

Attributes of Medicare beneficiaries with claims for coincident HIV or hepatitis infection and opioid-related harms have not been adequately described in the literature, and the factors associated with claims settled in 2015 for these infections or opioid overdose among beneficiaries are unknown. To address these knowledge gaps, we assessed (a) numbers and percentages of Medicare fee-for-service (FFS) beneficiaries with opioid-related claims or opioid overdose by age, sex, race/ethnicity, US census region of residence, and county of residence rated by vulnerability index [19] for IDU-related HIV and HCV outbreaks; (b) the association of claims for HIV infection and acute or chronic HBV and HCV infections with opioid-related claims; (c) factors associated with opioid overdose among beneficiaries with non-overdose opioid-related claims and with HIV, HBV, or HCV infection; and (d) factors associated with claims for heroin overdose among beneficiaries with non-overdose opioid-related claims.

## 2. Methods

### 2.1. Data Sources and Populations

We used the Medicare administrative enrollment and claims data [21] collected by CMS to estimate HIV, HBV, or HCV infections among beneficiaries who had FFS health insurance claims settled during 2015 (latest data availability year). We estimated the infection burden in 5 categories (Table A1): (a) HIV, (b) acute HBV, (c) chronic HBV, (d) acute HCV, and (e) chronic HCV; we also compared claims for ≥1 of these 5 infections with 0 claims. To identify these infections, we used diagnosis codes for beneficiaries who had ≥1 inpatient claim or ≥2 outpatient claims spaced ≥1 month apart [22]. The opioid-related and infection diagnoses were coded according to the International Classification of Diseases, 9th Revision, Clinical Modification (ICD-9-CM) [23] for the first three quarters of 2015 or the 10th Revision (ICD-10-CM) [24] for the fourth quarter.

We estimated numbers of beneficiaries with opioid-related claims for 2 diagnosis categories: (a) opioid abuse or opioid dependence and unspecified use (categorized and labelled opioid-related claims (ORCs)); and (b) opioid poisoning (categorized and labelled opioid overdose) (Table A2) [25]. Beneficiaries with ORCs had ≥1 inpatient claim or ≥2 outpatient claims spaced ≥1 month apart with ≥1 prespecified diagnosis code, and for opioid overdose, had ≥1 inpatient or outpatient claim with ≥1 prespecified diagnosis code. Beneficiaries with ORCs or opioid overdose were identified separately using a series of ICD-CM codes in multiple diagnosis fields in outpatient and inpatient clams based on the prespecified criteria. We classified each beneficiary as *Yes/No* for having or not having a claim for each category of opioid-related diagnosis or infection. We examined associations of ORCs, opioid overdose, and each infection by age group (≤64, 65–74, or ≥75 years), sex (male or female), race/ethnicity (non-Hispanic white, non-Hispanic black, Hispanic, Asian/Pacific Islander, American Indian/Alaska Native, or other/unknown), and US census region of residence (Northeast, West, Midwest, or South). We also estimated frequencies of persons with opioid-related claims residing in each county classified by vulnerability score (Vulnerable, the 5% of counties with the highest vulnerability, or Other) for HIV or HCV infections (scores are calculated for all US counties by assessing 15 indicators that can be associated with rapid dissemination of HIV or HCV infection through IDU) [19].

### 2.2. Analysis

We first analyzed demographic characteristics for beneficiaries with ORCs and calculated percentages of beneficiaries with or without each type of opioid-related claim among those who had or did not have HIV, HBV, or HCV claims. Second, we estimated the percentage of beneficiaries with claims for opioid overdose among those who had ORC claims. Third, we calculated percentages of persons with each type of opioid-related claim separately by demographic attributes of Medicare FFS enrollees who had or did not have claims for any of each category of selected infection. Fourth, we used multivariable logistic regression to examine the association of HIV, HBV, or HCV infection separately with ORC while controlling for age, sex, race/ethnicity, US census region of residence, and county-level vulnerability scores in the model. Fifth, we identified factors associated with claims for opioid overdose among beneficiaries with ORCs and compared the odds ratios of opioid overdose among beneficiaries with claims for each category of infection versus without infection. Similarly, we compared odds ratios of opioid overdose among beneficiaries with claims for ≥1 infection versus none of the 5 infections. Lastly, we examined associations of claims for heroin overdose with each infection among beneficiaries with ORCs. All analyses were performed by using SAS^®^ 9.4 and SAS Enterprise Guide^®^ 7.1 (SAS Institute, Inc., Cary, North Carolina) in the secured environment of the CMS Virtual Research Data Center through the Chronic Conditions Warehouse [26].

## 3. Results

### 3.1. Population Attributes of Beneficiaries with Concurrent Claims for Selected Infections and Opioid-Related Diagnoses

Of 40.6 million beneficiaries with FFS claims settled during 2015, 263,709 (0.6%) had ORCs, and 46,073 (0.1%) had claims for opioid overdose (Table 1). Compared with beneficiaries without opioid-related claims, those with claims had significantly different (*p* < 0.05) distributions by age group, sex, race/ethnicity, residence census region, and county vulnerability score. Among beneficiaries with ORCs, the majority were aged <75 years (89.3%), female (54.3%), non-Hispanic white (78.2%), residing in the South (45.2%), and not residing in vulnerable counties (93.6%). Similar differences in demographic distribution patterns were observed for beneficiaries with opioid overdose claims.

**Figure 1:**
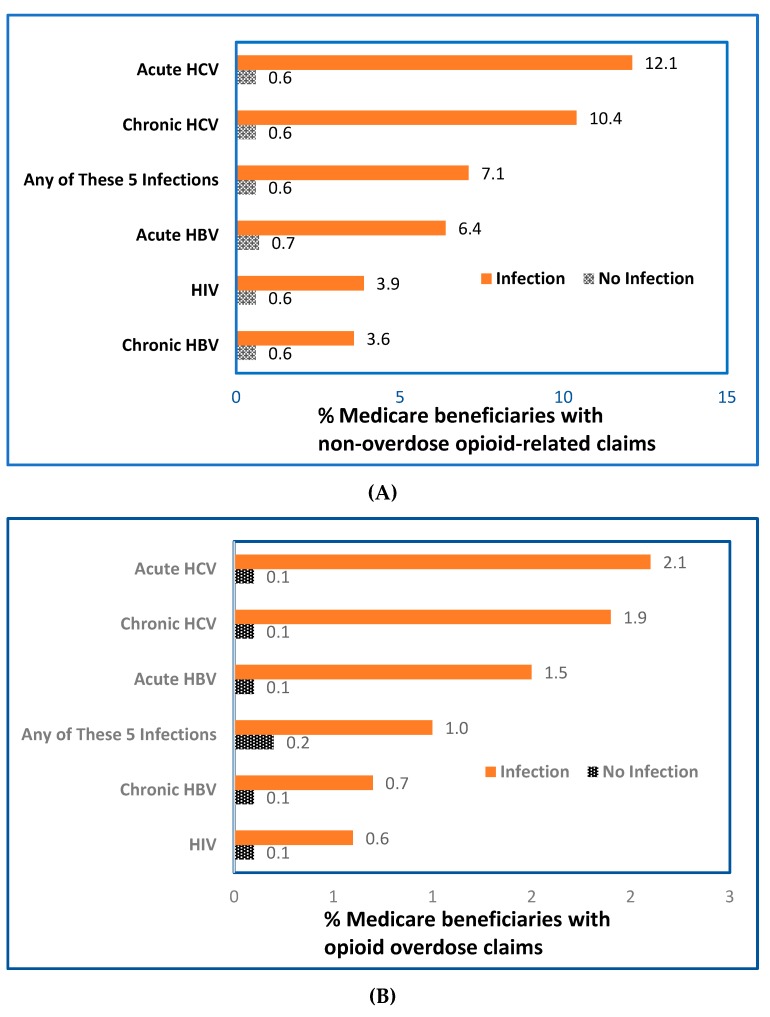
Percentage of Medicare beneficiaries with opioid-related claims among beneficiaries without (**A**) or with (**B**) claims for HIV, HBV, or HCV, United States, 2015; (*n* = 40.6 million beneficiaries with FFS claims for non-overdose opioid-related diagnoses). Abbreviations: FFS, fee-for service, HBV, hepatitis B virus; HCV, hepatitis C virus, HIV, human immunodeficiency virus. Data were calculated from 40.6 million beneficiaries with fee-for-service claims from Medicare administrative enrollment and claims data collected by the Centers for Medicare and Medicaid Services during 2015. Each beneficiary was classified as *Yes/No* for having or not having a claim for each category of opioid-related diagnoses or selected infection (HIV, HBV, or HCV infections). The bar labelled “Any of These 5 Infections” indicates that claims involved ≥1 of the 5 infections studied: HIV, acute or chronic HBV, and acute or chronic HCV.

Among beneficiaries with FFS claims, small percentages had claims for HIV infection (0.3%), acute HBV (0.05%), chronic HBV (0.06%), acute HCV (0.05%), chronic HCV (0.4%), or ≥1 of these 5 infections (0.8%) (Table S1 in the Supplement). Percentages of beneficiaries with opioid-related claims were significantly higher (*p* < 0.05) among those with claims for HIV, HBV, or HCV infection, compared with those without claims for the infections studied (Figure 1A; Table S1). The pattern observed for beneficiaries with claims for opioid overdose was similar to that observed for beneficiaries with ORCs (Figure 1B; Table S1). Among beneficiaries with opioid overdose claims, 32.7% also had ORCs (Table S2 in the Supplement).

Table 2 and Table S3 in the Supplement present the percentages of beneficiaries with ORCs or opioid overdose claims by demographics among beneficiaries with or without any of the 6 categories of infection. Among beneficiaries who had HIV claims, 3.9% had ORCs; this percentage increased to 12.1% among those who had claims for acute HCV infection (Table 2). The highest percentages of beneficiaries with ORC claims among enrollees with HIV claims were among persons aged ≤64 years (4.4%), female (4.5%), Hispanic (4.3%), and residing in the Northeast (6.5%) and in counties with a high vulnerability score. Among beneficiaries with HBV or HCV claims, the highest percentages of beneficiaries having ORCs were aged ≤64 years, male, non-Hispanic white, and residing in the Northeast and in vulnerable counties. For acute HCV, 17.1% having ORCs were aged ≤64 years versus <2% among those aged ≥75 years. The percentage of beneficiaries having ORCs was slightly higher among males than females among HBV-infected beneficiaries (acute, 7.2% for males versus 5.1% for females; chronic, 4.2% for males versus 2.8% for females). Similarly, males had a slightly higher percentage of ORCs among HCV-infected beneficiaries. Non-Hispanic whites with claims for acute and chronic HBV or chronic HCV had more frequent claims with diagnostic codes for ORCs than other racial/ethnic groups. The percentage of ORCs among beneficiaries who had claims for HIV infection was approximately the same among Hispanics and non-Hispanic whites. Asian/Pacific Islanders had the lowest percentage of ORCs than any other racial/ethnic group with HBV or HCV infections or ≥1 of the 5 infections versus none.

Except for acute HBV (highest percentage of claims in the South), beneficiaries residing in the Northeast had the highest ORCs among those who had claims for HIV, chronic HBV, acute or chronic HCV infections, or ≥1 of these 5 infections versus 0 infections (Table 2). Overall, beneficiaries residing in counties with the highest vulnerability scores had >2 times the ORCs than those who lived in other counties (1.4% versus 0.6%). This percentage was even higher among beneficiaries with claims for HIV infection (4.2%), acute HBV (20.0%), chronic HBV (12.3%), acute HCV (19.9%), chronic HCV (15.8%), or ≥1 of the 5 infections (13.5%) versus none of the infections.

Table S3 in the Supplement presents beneficiaries with claims for opioid overdose among those with HIV, HBV, or HCV infection claims. Among persons with claims for HIV, HBV, or HCV infection, the highest percentage of persons with opioid overdose was observed among those aged ≤64 years and non-Hispanic whites or American Indian/Alaska Natives. However, certain variations in the percentages of having a claim for opioid overdose were observed by demographics among those with claims for HIV, HBV, or HCV infection. For example, for persons who resided in counties with the highest vulnerability scores versus those who resided in other counties, the percentage of persons who had a claim for opioid overdose was higher among those with claims for acute HBV (3.7% versus 1.4%), chronic HCV (2.8% versus 1.8%), or ≥1 infection (2.3% versus 1.3%). However, this pattern was not observed for persons with claims for HIV and chronic HBV infection.

### 3.2. Association of Claims for Selected Infections with Opioid-Related Claims

A statistically significant association of having a claim for HIV, HBV, or HCV infection was observed among beneficiaries with ORCs after controlling for age, sex, race/ethnicity, US region of residence, and county-level vulnerability (Table 3). The odds of having a claim for HIV among beneficiaries with ORCs was more than twice (adjusted odds ratio (aOR), 2.3; 95% confidence interval (CI), 2.3–2.4; *p* < 0.05) that of those who did not have an ORC. This significant association was remarkably stronger among beneficiaries with claims for ORCs and acute HBV infection (aOR, 6.7; 95% CI, 6.3–7.1), chronic HBV (aOR, 5.0; 95% CI, 4.7–5.4), acute HCV infection (aOR, 9.6; 95% CI, 9.2–10.0), or chronic HCV infection (aOR, 8.9; 95% CI, 8.7–9.1). Beneficiaries with ORCs had approximately 6 times the odds of having a claim for ≥1 of the 5 infections, compared with those who did not (aOR, 5.8; 95% CI, 5.7–5.9). Beneficiaries with ORCs with the selected demographic characteristics in this study were also significantly (*p* < 0.0001) associated with having a claim for these 6 infection categories.

### 3.3. Factors Associated with Opioid Overdose among Beneficiaries with Opioid-Related Claims

Beneficiaries with claims for ORCs and HIV, HBV, or HCV infection had a 1.1–1.9-fold odds of having a claim for opioid overdose, compared with those who did not (Table 4). By examining claims involving coinfection with ≥1 of the 5 infections among beneficiaries with ORCs, beneficiaries with ≥1 HIV, HBV, or HCV infection had twice the odds (aOR, 2.0; 95% CI, 1.9–2.1) of having claims for opioid overdose, compared with those who had none of these infections (Table S4 in the Supplement). A similar pattern of association between claims for heroin overdose with bloodborne infections was observed, but its association was even stronger for acute (aOR, 2.4; 95% CI, 1.8–3.0) or chronic HBV (aOR, 1.4; 95% CI, 1.0–2.0), or acute (aOR, 1.5; 95% CI, 1.2–1.8) or chronic HCV (aOR, 2.7; 95% CI, 2.5–3.0) (Table S5 in the Supplement).

## 4. Discussion

Among 40.6 million beneficiaries with FFS claims during 2015, 0.6% had non-overdose ORCs and 0.1% had opioid overdose claims. Among beneficiaries with opioid overdose claims, 32.7% also had ORCs. Beneficiaries with ORCs were substantially more likely than those without ORCs to be aged <75 years, female, non-Hispanic white, residents of the US South, and residents of counties less vulnerable to IDU-related HIV and HCV outbreaks. Beneficiaries with ORCs were more likely to have claims for HIV (2.3 times), acute HBV (6.7 times), chronic HBV (5.0 times), acute HCV (9.6 times), chronic HCV (8.9 times), or any 1 of those infections (5.8 times), when adjusted for age, sex, race/ethnicity, region of residence, and residence in counties with a high vulnerability index. Factors associated with opioid overdose included claims for acute HBV, acute HCV, and chronic HCV, residence in a county with a high vulnerability score, residence in the US Midwest, other/unknown race/ethnicity, and age <75 years.

Our findings are consistent with recent evidence reported in previous studies about opioid use (e.g., 43.7% of disabled beneficiaries aged ≤64 years reported prescription opioid use during 2011) [27]. Our data reveal that the highest percentage of beneficiaries with claims for ORCs and acute HBV infection resided in the South, but higher percentages of beneficiaries with claims for HIV, HCV, or chronic HBV infection resided in the Northeast. Recently, CDC reported that opioid overdose treated in emergency departments increased approximately 30% in the majority of states, with a peak of 70% in the Midwest during the 14 months before September 2017 [28]. Moreover, opioid prescribing increased for men and women in all age groups and in all census regions, but varied by states with rural/urban differences [29,30,31].

Our results extend previous studies and confirm that ORCs are strongly associated with the 6 categories of infection (i.e., HIV [32], acute or chronic HBV [33], and acute or chronic HCV [13]) [34,35]. Beneficiaries with claims for opioid-related diagnoses and acute HBV or acute or chronic HCV infection were 1–2 times as likely to have opioid overdose claims, compared with beneficiaries without these claims. For heroin overdoses, the association was even stronger, with the odds 1–3 times higher, compared with beneficiaries without the 5 infection claims. However, we did not observe claims for HIV infection to be independently associated with opioid overdose nor heroin overdose among beneficiaries with ORCs after adjusting for demographics and HBV or HCV infection.

Other studies have reported that patients with HIV infection are more likely to be prescribed opioids than uninfected persons [36,37], have an increased risk for opioid-related death [38,39], and have a higher prevalence of risk factors associated with major opioid-related adverse events (e.g., mental health or alcohol and substance use disorders) [40]. Correlation of high-dose opioid receipt was reported with HIV infection among aging patients [32,41]. Opioid use disorder has been reported to impair immune function [42], interact with antiretroviral agents in treatment of persons coinfected with HIV and HCV [35,43,44], and increase readmission rates and health care usage after surgery [45].

Our findings indicate a strong association between ORCs and HIV, an association that is even stronger among beneficiaries with HCV or HBV claims. For beneficiaries with ORCs, the odds ratios range from 5.0 for having claims for chronic HBV infection to 9.5 for having claims for acute HCV infection, compared with beneficiaries without ORCs; this range is more than the odds ratio of having claims for HIV alone (i.e., aOR, 2.3). A systematic review of viral hepatitis among injection-drug users estimated that worldwide, approximately 10.0 million and 6.4 million persons who inject drugs might have HCV antibody (anti-HCV) and total hepatitis B core antibody, respectively, and concluded that the prevalence of anti-HCV among persons who inject drugs is far greater than that of HIV [46]. Recently, HIV and viral hepatitis coinfection has become increasingly recognized as a major public health concern [47].

CDC reports that approximately one third of persons with HIV infection are coinfected with either HBV or HCV in the United States [47]. Additionally, hepatitis B vaccination rates among persons who inject drugs are lower than those among the general population [48], and no hepatitis C vaccine is available [7,49]. Hence, an urgent need exists to increase hepatitis B vaccination coverage and to develop a hepatitis C vaccine for the general population; this need is especially crucial for persons who inject drugs.

Our results indicate that beneficiaries with ORCs and HIV, HBV, or HCV infections are more likely to have a claim for opioid or heroin overdose, compared with those without these infections. A prior study reported that opioid overdose deaths were high among those aged 25–64 years and death rates among non-Hispanic whites were 4 times higher than among Hispanic or black populations [50]. A recent study reported higher average inpatient stays and emergency department visits among patients aged ≥65 years when comparing opioid-related inpatient stays and nonopioid-related stays during 2015 [16].

## 5. Strengths and Limitations

Strengths of this study include the large population-based samples from Medicare data for 98% of the US population aged ≥65 years and also beneficiaries of all ages with qualifying conditions (e.g., end-stage renal disease). The majority of published studies have focused on opioid-related experiences and bloodborne infections primarily among young populations [51]. Our study allows for detailed analysis of opioid-related diagnoses and bloodborne infections among older adults or persons with disabilities who are more likely than younger adults to have adverse effects associated with opioid use [16].

Our findings are subject to certain limitations. First, this is a cross-sectional analysis of data for beneficiaries with claims for opioid-related diagnoses and selected bloodborne infections; therefore, a temporal association between persons with ORCs and the studied infections cannot be established. Second, this study used both ICD-9-CM and ICD-10-CM codes, and lack of diagnostic codes aligning directly with current clinical diagnostic criteria presents challenges. In our study, diagnostic codes were used to identify claims reflecting a broad spectrum of opioid use. Clinical terminology for certain opioid-related harms has varied over time [52]. For example, such terms as abuse are avoided to reduce stigma [53] but remain in diagnostic codes that do not clearly align with clinical diagnostic criteria [54]. Opioid use disorder, as defined by the Diagnostic and Statistical Manual of Mental Disorders (DSM-5) is “a problematic pattern of opioid use leading to clinically significant impairment or distress [55] (page 541)” characterized by patients having ≥2 of 11 defined criteria (e.g., craving or strong desire or urge to use) within a 12-month interval [55]. Opioid misuse has been defined as including either the use of an illicit opioid or the nonmedical use of prescribed opioids [15].

An analysis of Medicare claims excluding diagnostic codes (e.g., for uncomplicated, unspecified opioid use) might reveal stronger associations of the selected infections with codes that clinicians might use for such opioid-related harms as opioid use disorder. The effects of the ICD-9-CM to ICD-10-CM transition in 2015 on the 2015 Medicare claims pertaining to opioid-related diagnoses and bloodborne infections were not assessed. A recent study examining trends in opioid-related inpatient stays using 2015 Healthcare Cost and Utilization Project data reported that 14.1% of the estimated opioid-related hospital stays were attributable to the transition to ICD-10-CM from ICD-9-CM in 2015 [56]. Therefore, our estimates might have been similarly affected. Third, our analysis used administrative claims data on infections that are underdiagnosed; therefore, the prevalence of HBV, HCV, or HIV are likely underestimated in this study. CDC recently examined National Health and Nutrition Examination Survey data and estimated that 0.38 million HCV antibody-positive persons were not included in the survey’s sampling frame during 2013–2016 [57]. Fourth, our analysis included beneficiaries with these bloodborne infections based on diagnosis codes in claims data. We could not determine whether infections were new or chronic. Fifth, this study used diagnosis codes to define opioid-related claims and opioid overdose ignoring whether or not the claim requested reimbursement for those conditions. Sixth, our study examined the association while adjusting for county-level composite index or vulnerability scores. This index contains 15 county-level indicators associated with HIV or acute HCV infection [19]. We hypothesized that all beneficiaries in the same county had uniform socioeconomic attributes and ignored variations in other social factors or lifestyle changes among and between persons that might have influenced the selected health outcomes. Finally, this study did not account for other risk factors that have been correlated with opioid overdose (e.g., comorbid psychiatric and medical disorders) [58].

## 6. Conclusions

We observed small percentages of FFS beneficiaries with ORCs and with claims for HIV, HBV, or HCV infection. Strong, independent, and statistically significant factors associated with claims for opioid overdose or each of the bloodborne infections included ORCs and selected demographic attributes. These results can help guide interventions intended to reduce incidences of HIV, HCV, or HBV infections among Medicare beneficiaries with ORCs.

## Figures and Tables

**Table 1:** Percentage of Medicare fee-for-service beneficiaries with and without claims ^a^ for opioid-related diagnoses, by demographic attributes, United States, 2015.

Characteristic	Non-Overdose Opioid-Related Claim ^b,c^		Opioid Overdose Claim ^b,c^		Total
	Yes	No	Yes	No	
	0.65% (*n* = 263,709)	99.35% (*n* = 40.3M)	0.11% (*n* = 46,073)	99.89% (*n* = 40.5M)	100.00% (*n* = 40.6M)
**Age (years), %**					
≤64	66.7	16.7	58.4	17.0	17.1
65–74	22.6	47.1	25.4	47.0	47.0
≥75	10.7	36.2	16.2	36.0	36.0
**Sex**					
Male	45.7	46.6	42.0	46.6	46.6
Female	54.3	53.4	58.0	53.4	53.4
**Race/ethnicity, %**					
Non-Hispanic white	78.2	77.6	79.4	77.6	77.6
Non-Hispanic black	13.3	9.9	12.1	9.9	9.9
Hispanic	5.8	7.0	5.6	7.0	7.0
Asian/Pacific Islander	0.6	2.9	0.7	2.9	2.9
American Indian/Alaska Native	1.0	0.6	1.1	0.6	0.6
Other/unknown	1.1	2.2	1.1	2.1	2.1
**US census region of residence, %**					
Northeast	19.7	18.6	18.1	18.6	18.6
West	18.6	19.2	18.5	19.2	19.2
Midwest	16.5	22.0	21.8	22.0	22.0
South	45.2	38.4	41.6	38.5	38.5
**County of residence by vulnerability score ^d^**					
Highest vulnerability	6.4	3.0	4.3	3.0	3.0
Other	93.6	97.0	95.7	97.0	97.0

M, million. ^a^ Diagnosis code used for ≥1 inpatient claim or ≥2 outpatient claims spaced ≥1 month apart.; ^b^ Significantly different distributions by demographics at a 2-tailed Chi-square test with *p* < 0.05; ^c^ Each beneficiary was classified as *Yes/No* for having or not having a claim for each category of opioid-related diagnoses or opioid overdose; ^d^ County vulnerability scores were reported by Van Handel et al in 2016. The top 5% of counties with the highest vulnerability scores were classified as *Highest vulnerability*, and the remaining 95% of counties were classified as *Other.*

**Table 2:** Number and percentage of beneficiaries with opioid-related claims, ^a^ by demographic characteristics among the Medicare fee-for-service beneficiaries who had or did not have HIV, HBV, or HCV claims, ^a,b^ United States, 2015.

	HIV		Acute HBV		Chronic HBV		Acute HCV		Chronic HCV		These 5 Infections		Total
Infection Status	Yes	No	Yes	No	Yes	No	Yes	No	Yes	No	≥1 Infection	None of These 5 Infections	
Number of beneficiaries	119 312	40.4M	19 553	40.5M	23 671	40.5M	20 322	40.5M	165 159	40.4M	311 657	40.3M	40.6M
Percentage (number) of beneficiaries with Opioid-Related Claims	3.90 (*n* = 4655)	0.64 (*n* = 259,054)	6.42 (*n* = 1255)	0.65 (*n* = 262, 454)	3.64 (*n* = 861)	0.65 (*n* = 262, 848)	12.13 (*n* = 2466)	0.64 (*n* = 261,243)	10.42 (*n* = 17,214)	0.61 (*n* = 246,495)	7.09 (*n* = 22,111)	0.60 (*n* = 241,598)	0.65 (*n* = 263,709)
**Characteristic**													
**Age (years), %**													
≤64	4.4	2.5	11.9	2.5	7.9	2.5	17.1	2.5	14.6	2.4	9.4	2.4	2.55
65–74	2.6	0.3	2.9	0.3	1.9	0.3	6.5	0.3	5.9	0.3	4.3	0.3	0.31
≥75	0.6	0.4	0.9	0.4	0.5	0.4	1.8	0.4	1.8	0.4	1.3	0.4	0.35
**Sex, %**													
Male	3.7	0.6	7.2	0.6	4.2	0.6	12.8	0.6	11.0	0.6	7.1	0.6	0.6
Female	4.5	0.7	5.1	0.7	2.8	0.7	11.2	0.7	9.5	0.6	7.2	0.6	0.7
**Race/ethnicity, %**													
Non-Hispanic white	4.1	0.7	9.7	0.7	7.3	0.7	13.8	0.7	11.9	0.6	9.0	0.6	0.7
Non-Hispanic black	3.9	0.8	5.9	0.9	5.0	0.9	9.5	0.9	8.0	0.8	5.2	0.8	0.9
Hispanic	4.3	0.5	0.5	0.5	4.7	0.5	11.8	0.5	11.0	0.5	7.0	0.5	0.5
Asian/Pacific Islander	1.8	0.1	0.2	0.1	0.1	0.1	2.4	0.1	1.6	0.1	0.7	0.1	0.1
American Indian/Alaska Native	1.6	1.2	7.8	1.2	4.7	1.2	14.5	1.2	11.6	1.1	9.0	1.1	1.2
Other/unknown	1.2	0.3	2.0	0.3	0.8	0.3	9.9	1.1	7.0	0.3	3.9	0.3	0.3
**US census region, by state, %**													
Northeast	6.5	0.7	6.9	0.7	4.9	0.7	16.5	0.7	15.6	0.6	10.3	0.6	0.7
West	3.1	0.6	4.1	0.6	1.9	0.6	9.5	0.6	9.1	0.6	6.2	0.6	0.6
Midwest	3.3	0.5	6.8	0.5	4.6	0.5	11.8	0.5	9.4	0.5	6.9	0.5	0.5
South	3.1	0.8	7.4	0.8	4.5	0.8	11.1	0.8	8.8	0.7	6.0	0.7	0.8
**County vulnerability score ^c^, %**													
Highest vulnerability	4.2	1.4	20.0	1.4	12.3	1.4	19.9	1.4	15.8	1.4	13.5	1.4	1.4
Other	3.9	0.6	6.0	0.6	3.5	0.6	11.9	0.6	10.3	0.6	7.0	0.6	0.6

Abbreviations: HBV, hepatitis B virus; HCV, hepatitis C virus; M, million. ^a^ Diagnosis code used ≥1 inpatient claim or ≥2 outpatient claims spaced ≥1 month apart. ^b^ Each beneficiary was classified as *Yes/No* for having or not having a claim for each category of opioid-related diagnoses or selected infection (HIV, HBV, or HCV infections). The table label “Any of these of infections” indicates claims involved ≥1 of the 5 infections studied: HIV, acute or chronic HBV, and acute or chronic HCV. ^c^ County vulnerability scores were reported by Van Handel et al in 2016. The top 5% of counties with the highest vulnerability scores were classified as *Highest vulnerability*, and the remaining 95% of counties were classified as *Other*.

**Table 3:** Adjusted odds ratios (aORs) and 95% confidence intervals (CIs) for association of claims for HIV, HBV, or HCV infections with non-overdose opioid-related claims or each demographic attribute of Medicare beneficiaries, United States, 2015.

Risk Factors	HIV Infection		Acute HBV Infection		Chronic HBV Infection		Acute HCV Infection		Chronic HCV Infection		Any of These 5 Infections	
	aOR	95% CI	aOR	95% CI	aOR	95% CI	aOR	95% CI	aOR	95% CI	aOR	95% CI
**Age (years)**												
≤64	26.1	25.3–26.8 ^a^	3.7	3.6–3.9 ^a^	3.2	3.1–3.3 ^a^	8.4	8.0–8.8 ^a^	9.3	9.1–9.4 ^a^	10.4	10.3–10.5 ^a^
65–74	3.2	3.10–3.3 ^a^	1.3	1.2–1.3 ^a^	1.3	1.3–1.4 ^a^	2.2	2.1–2.3 ^a^	2.6	2.5–2.6 ^a^	2.2	2.2–2.3 ^a^
≥75	Ref	Ref	Ref	Ref	Ref	Ref	Ref	Ref	Ref	Ref	Ref	Ref
**Sex**												
Male	2.9	2.8–2.9 ^a^	1.7	1.7–1.8 ^a^	1.7	1.7–1.8 ^a^	1.5	1.5–1.6 ^a^	1.6	1.5–1.6 ^a^	1.9	1.9–1.9 ^a^
Female	Ref	Ref	Ref	Ref	Ref	Ref	Ref	Ref	Ref	Ref	Ref	Ref
**Race/ethnicity**												
Non-Hispanic white	Ref	Ref	Ref	Ref	Ref	Ref	Ref	Ref	Ref	Ref	Ref	Ref
Non-Hispanic black	4.5	4.4–4.6 ^a^	3.1	3.0–3.3 ^a^	3.3	3.2–3.5 ^a^	2.3	2.2–2.4 ^a^	2.3	2.3–2.4 ^a^	3.1	3.1–3.1 ^a^
Hispanic	2.4	2.3–2.4 ^a^	1.7	1.6–1.8 ^a^	1.7	1.6–1.8 ^a^	1.6	1.5–1.7 ^a^	1.5	1.4–1.5 ^a^	1.7	1.7–1.8 ^a^
Asian/Pacific Islander	0.7	0.7–0.8 ^a^	13.8	13.2–14.3 ^a^	32.7	31.7–33.8 ^a^	1.5	1.4–1.6 ^a^	1.4	1.4–1.5 ^a^	3.3	3.2–3.3 ^a^
American Indian/Alaska Native	1.1	1.0–1.2 ^a^	1.2	1.0–1.5 ^a^	1.3	1.1–1.6 ^a^	1.2	1.0–1.4 ^a^	1.4	1.3–1.4 ^a^	1.3	1.2–1.3 ^a^
Other/unknown	1.0	0.9–1.0	2.1	2.0–2.3 ^a^	3.5	3.3–3.8 ^a^	0.7	0.6–0.8 ^a^	0.7	0.7–0.7 ^a^	1.0	1.0–1.0
**US census region**												
Northeast	Ref	Ref	Ref	Ref	Ref	Ref	Ref	Ref	Ref	Ref	Ref	Ref
Midwest	0.5	0.5–0.5 ^a^	0.8	0.7–0.8 ^a^	0.6	0.5–0.6 ^a^	0.6	0.6–0.6 ^a^	0.7	0.7–0.7 ^a^	0.6	0.6–0.6 ^a^
South	0.8	0.8–0.8 ^a^	0.8	0.8–0.8 ^a^	0.7	0.7–0.7 ^a^	0.7	0.7–0.7 ^a^	0.8	0.8–0.8 ^a^	0.8	0.8–0.8 ^a^
West	0.8	0.8–0.8 ^a^	0.8	0.8–0.8 ^a^	1.0	1.0–1.1	0.9	0.9–1.0 ^a^	1.2	1.2–1.2 ^a^	1.0	1.0–1.0
**County vulnerability score ^b^**												
Highest vulnerability	2.5	2.4–2.7 ^a^	0.8	0.7–0.8 ^a^	0.8	0.8–0.9 ^a^	0.9	0.9–1.0	1.2	1.2–1.3 ^a^	1.4	1.4–1.4 ^a^
Other	Ref	Ref	Ref	Ref	Ref	Ref	Ref	Ref	Ref	Ref	Ref	Ref
**Opioid-related claims (main predictor variable)**												
Yes	2.3	2.3–2.4 ^a^	6.7	6.3–7.1 ^a^	5.0	4.7–5.4 ^a^	9.6	9.2–10.0 ^a^	8.9	8.7–9.1 ^a^	5.8	5.7–5.9 ^a^
No	Ref	Ref	Ref	Ref	Ref	Ref	Ref	Ref	Ref	Ref	Ref	Ref

Abbreviations: HBV, hepatitis B virus; HCV, hepatitis C virus; Ref, reference. ^a^ Significant for Chi-square test at *p* < 0.05. ^b^ County vulnerability scores were reported by Van Handel et al in 2016. The top 5% of counties with the highest vulnerability scores were classified as *Highest vulnerability*, and the remaining 95% of counties were classified as *Other.*

**Table 4:** Adjusted odds ratios (aORs) and 95% confidence intervals (CIs) for association of opioid overdose claims with each demographic attribute of Medicare beneficiaries with claims for opioid-related diagnoses, United States, 2015.

Characteristic	Opioid Overdose	
	aOR	95% CI
**Age (years)**		
≤64	1.9	1.7–2.0 ^a^
65–74	1.5	1.4–1.7 ^a^
≥75	Ref	Ref
**Sex**		
Male	1.0	0.9–1.0
Female	Ref	Ref
**Race/ethnicity**		
Non-Hispanic white	Ref	Ref
Non-Hispanic black	0.8	0.8–0.9 ^a^
Hispanic	0.9	0.9–1.0 ^a^
Asian/Pacific Islander	0.8	0.6–1.0
American Indian/Alaska Native	1.1	0.9–1.2
Other/unknown	1.2	1.1–1.2 ^a^
**US census region**		
Northeast	Ref	Ref
Midwest	1.2	1.1–1.2 ^a^
South	0.8	0.7–0.8 ^a^
West	0.9	0.9–1.0 ^a^
**County vulnerability score ^b^**		
Highest vulnerability	1.4	1.3–1.6 ^a^
Other	Ref	Ref
**Infection (primary predictor variable)**		
**HIV**		
Yes	1.1	1.0–1.2
No	Ref	Ref
**Acute HBV**		
Yes	1.9	1.6–2.3 ^a^
No	Ref	Ref
**Chronic HBV**		
Yes	1.2	1.0–1.5
No	Ref	Ref
**Acute HCV**		
Yes	1.3	1.2–1.5 ^a^
No	Ref	Ref
**Chronic HCV**		
Yes	1.9	1.8–2.0 ^a^
No	Ref	Ref

Abbreviations: HBV, hepatitis B virus; HCV, hepatitis C virus; Ref, reference. ^a^ Significant for Chi-square test at *p* < 0.05. ^b^ County vulnerability scores were reported by Van Handel et al in 2016. The top 5% of counties with the highest vulnerability scores were classified as *Highest vulnerability*, and the remaining counties were classified as *Other*.

## Supplementary Materials

The following are available online at https://www.mdpi.com/2077-0383/8/11/1768/s1. Table S1: Number and Percentage of Medicare Fee-for-Service Beneficiaries With or Without Opioid-related Claims Among Those Who Had Claims for HIV, HBV, or HCV, United States, 2015; Table S2: Number and Percentage of Medicare Fee-for-Service Beneficiaries Who Had Claims for Opioid-related Diagnoses, by Those with Claims for Opioid Overdose, United States, 2015; Table S3: Number and Percentage of Beneficiaries with Opioid Overdose Claims,^a^ by Demographic Characteristics Among the Medicare Fee-for-Service Beneficiaries Who Had or Did Not Have HIV,^b^ HBV, or HCV ^b^ Claims, United States, 2015; Table S4: Adjusted Odds Ratios (aORs) and 95% Confidence Intervals (CIs) for Association of Opioid Overdose Claims with ≥1 HIV, HBV, or HCV Infection Among Medicare Beneficiaries with Non-overdose Opioid-related Claims, United States, 2015; Table S5: Adjusted Odds Ratios (ORs) and 95% Confidence Intervals (CIs) for Association of Heroin Overdose Claims Among Medicare Beneficiaries With Non-overdose Opioid-related Claims, United States, 2015.

## Appendix A

**Table A1:** International Classification of Diseases, 9th Revision, Clinical Modification (ICD-9-CM) and International Classification of Diseases, 10th Revision, Clinical Modification (ICD-10-CM) Codes for HIV, HBV and HCV Infection Used in the Study.

Disease	ICD-9-CM Code	ICD-10-CM Code
HIV/AIDS	042, V08, 079.53, or 795.71	B20, Z21, B97.35, or R75
Hepatitis B		
Acute	070.20, 070.21, 070.30,070.31, or 070.52	B16
Chronic	070.22, 070.23, 070.32, 070.33, or 070.42	B17.0, B180.0, or B18.1
Hepatitis C		
Acute	070.41 or 070.51	B17.1
Chronic	070.44 or 070.54	B18.2

Abbreviations: HBV, hepatitis B virus; HCV, hepatitis C virus.

## Appendix B

**Table A2:** International Classification of Diseases, 9th Revision, Clinical Modification (ICD-9-CM) and International Classification of Diseases, 10th Revision, Clinical Modification (ICD-10-CM) Codes for Opioid-Related Claims ^a^ and Heroin Overdose Used in the Study.

Condition	ICD-9-CM Code	ICD-10-CM Code
Opioid abuse	30550, 30551, 30552	F1110, F11120, F11121, F11122, F11129, F1114, F11150, F11151, F11159, F11181, F11182, F11188, F1119
Opioid dependence and unspecified use	30400, 30401, 30402, 30470, 30471, 30472	F1120, F11220, F11221, F11222, F11229, F1123, F1124, F11250, F11251, F11259, F11281, F11282, F11288, F1129, F1190, F11920, F11921, F11922, F11929, F1193, F1194, F11950, F11951, F11959, F11981, F11982, F11988, F1199
Opioid poisoning	96500, 96501, 96502, 96509, 9701, E8500, E8501, E8502	T400X1A, T400X1D, T400X1S, T400X1S, T400X4A, T400X4D, T400X4S, T401X1A, T401X1D, T401X1S, T401X4A, T401X4D, T401X4S, T402X1A, T402X1D, T402X1S, T402X4A, T402X4D, T402X4S, T403X1A, T403X1D, T403X1S, T403X4A, T403X4D, T403X4S, T404X1A, T404X1D, T404X1S, T404X4A, T404X4D, T404X4S, T40601A, T40601D, T40601S, T40604A, T40604D, T40604S, T40691A, T40691D, T40691S, T40694A, T40694D, T40694S
Heroin poisoning	96501, E8500	T40.1X1x–1X4x

^a^ Source: Moore BJ, Barrett ML. Case Study: Exploring How Opioid-Related Diagnosis Codes Translate From ICD-9-CM to ICD-10-CM. Rockville, MD: US Agency for Healthcare Research and Quality; 2018.
