# Supplementary material for: Opioid-Related Diagnoses and Concurrent Claims for HIV, HBV, or HCV among Medicare Beneficiaries, United States, 2015
Source: J Clin Med. 2019 Oct 24;8(11):1768. doi: 10.3390/jcm8111768 (PMC6912616; doi:10.3390/jcm8111768)
Supplement: Supplementary file 1 [file jcm-08-01768-s001.pdf]

**Table S1.** Number and Percentage of Medicare Fee-for-Service Beneficiaries With or Without Opioid-related Claims Among Those Who Had Claims for HIV, HBV, or HCV, United States, 2015.

| Attribute | HIV [c] | | Acute HBV [c] | | Chronic HBV [c] | | Acute HCV [c] | | Chronic HCV [c] | | These 5 Infections [c] | | Total |
|---|---|---|---|---|---|---|---|---|---|---|---|---|---|
| | Yes | No | Yes | No | Yes | No | Yes | No | Yes | No | ≥1 Infection | None of These 5 Infections | |
| | No. (%) | No. (%) | No. (%) | No. (%) | No. (%) | No. (%) | No. (%) | No. (%) | No. (%) | No. (%) | No. (%) | No. (%) | No. (%) |
| **Opioid-related diagnoses [a]** | | | | | | | | | | | | | |
| Yes | 4655 (3.9) | 259,054 (0.6) | 1255 (6.4) | 262,454 (0.7) | 861 (3.6) | 262,848 (0.6) | 2466 (12.1) | 261,243 (0.6) | 17,214 (10.4) | 246,495 (0.6) | 22,111 (7.1) | 241,598 (0.6) | 263,709 (0.6) |
| No | 114,657 (96.1) | 40.2M (99.4) | 18,298 (93.6) | 40.3M (99.3) | 22,810 (96.4) | 40.3M (99.4) | 17,858 (87.9) | 40.3M (99.4) | 147,945 (89.6) | 40.2M (99.4) | 289,546 (92.9) | 40.0M (99.4) | 40.3M (99.4) |
| **Opioid overdose [b]** | | | | | | | | | | | | | |
| Yes | 762 (0.6) | 45,311 (0.1) | 287 (1.5) | 45,786 (0.1) | 176 (0.7) | 45,897 (0.1) | 420 (2.1) | 45,653 (0.1) | 3080 (1.9) | 42,993 (0.1) | 186 (1.0) | 79,960 (0.2) | 46,073 (0.1) |
| No | 118,550 (99.4) | 40.4M (99.9) | 19,266 (98.5) | 40.5M (99.9) | 23,495 (99.3) | 40.5M (99.9) | 19,902 (97.9) | 40.5M (99.9) | 162,079 (98.1) | 40.4M (99.9) | 19,367 (99.0) | 40.2M (99.8) | 40.5M (99.9) |
| Total | 119,312 (0.3) | 40.4M (99.7) | 19,553 (0.05) | 40.5M (99.9) | 23,671 (0.06) | 40.5M (99.9) | 20,322 (0.05) | 40.5M (99.9) | 165,159 (0.4) | 40.4M (99.6) | 311,657 (0.8) | 40.3M (99.2) | 40.6M (100.0) |

Abbreviations: HBV, hepatitis B virus; HCV, hepatitis C virus, M, million. [a] Defined as ≥1 diagnosis code in the inpatient claims or ≥2 outpatient claims spaced ≥1 month apart. [b] Defined as ≥1 diagnosis code in the inpatient or outpatient claim. [C] Significant difference in percentage of opioid-related claims between beneficiaries with and without claims for HIV, HBV, or HCV at a 2-tailed chi-square test with $p < 0.05$.

**Table S2.** Number and Percentage of Medicare Fee-for-Service Beneficiaries Who Had Claims for Opioid-related Diagnoses, by Those with Claims for Opioid Overdose, United States, 2015.

| Opioid-related Claims | Opioid Overdose [b] | | Total |
|---|---|---|---|
| | Yes | No | |
| | No. (%) | No. (%) | No. (%) |
| **Yes** | 15,076 (32.7) | 248,633 (0.6) | 263,709 (0.6) |
| **No** | 30,997 (67.3) | 40.3M (99.4) | 40.3M (99.4) |
| **Total** | 46,073 (0.1) | 40.5M (99.9) | 40.6M (100.0) |

[a] Defined as ≥1 diagnosis code in the inpatient claims or ≥2 outpatient claims spaced ≥1 month apart. [b] Defined as ≥1 diagnosis code in the inpatient or outpatient claim.

**Table S3.** Number and Percentage of Beneficiaries with Opioid Overdose Claims,[a] by Demographic Characteristics Among the Medicare Fee-for-Service Beneficiaries Who Had or Did Not Have HIV,[b] HBV, or HCV [b] Claims, United States, 2015.

| Infection Status | HIV | | Acute HBV | | Chronic HBV | | Acute HCV | | Chronic HCV | | These 5 Infections | | Total |
|---|---|---|---|---|---|---|---|---|---|---|---|---|---|
| | Yes | No | Yes | No | Yes | No | Yes | No | Yes | No | ≥1 Infection | None of These 5 Infections | |
| **Number of Beneficiaries** | 119,312 | 40.4M | 19,553 | 40.5M | 23,671 | 40.5M | 20,322 | 40.5M | 165,159 | 40.4M | 311,657 | 40.3M | 40.6M |
| **Percentage (number) of Beneficiaries with Opioid Overdose Claims** | **0.64 (*n* = 762)** | **0.11 (*n* = 45,311)** | **1.47 (*n* = 287)** | **0.11 (*n* = 45,786)** | **0.74 (*n* = 176)** | **0.11 (*n* = 45,897)** | **2.07 (*n* = 420)** | **0.11 (*n* = 45,653)** | **1.86 (*n* = 3080)** | **0.11 (*n* = 42,993)** | **1.27 (*n* = 3954)** | **0.10 (*n* = 42,119)** | **0.11% (*n* = 46,073)** |
| **Characteristic** | | | | | | | | | | | | | |
| **Age (years), %** | | | | | | | | | | | | | |
| ≤64 | 0.7 | 0.4 | 2.7 | 0.4 | 1.5 | 0.4 | 2.9 | 0.4 | 2.6 | 0.4 | 1.7 | 0.4 | 0.4 |
| 65-74 | 0.4 | 0.1 | 0.6 | 0.1 | 0.4 | 0.1 | 1.0 | 0.1 | 1.0 | 0.1 | 0.7 | 0.1 | 0.1 |
| ≥75 | 0.2 | 0.1 | 0.3 | 0.1 | 0.2 | 0.1 | 0.4 | 0.1 | 0.6 | 0.1 | 0.4 | 0.1 | 0.1 |
| **Sex** | | | | | | | | | | | | | |
| Male | 0.6 | 0.1 | 1.5 | 0.1 | 0.8 | 0.1 | 2.1 | 0.1 | 1.9 | 0.1 | 1.2 | 0.1 | 0.1 |
| Female | 0.7 | 0.1 | 1.4 | 0.1 | 0.7 | 0.1 | 2.0 | 0.1 | 1.8 | 0.1 | 1.4 | 0.1 | 0.1 |
| **Race/ethnicity, %** | | | | | | | | | | | | | |
| Non-Hispanic white | 0.8 | 0.1 | 2.4 | 0.1 | 1.6 | 0.1 | 2.5 | 0.1 | 2.2 | 0.1 | 1.7 | 0.1 | 0.1 |
| Non-Hispanic black | 0.6 | 0.1 | 1.0 | 0.1 | 0.8 | 0.1 | 1.3 | 0.1 | 1.3 | 0.1 | 0.8 | 0.1 | 0.1 |
| Hispanic | 0.5 | 0.1 | 1.7 | 0.1 | 0.7 | 0.1 | 1.9 | 0.1 | 1.6 | 0.1 | 1.0 | 0.1 | 0.1 |
| Asian/Pacific Islander | 0.3 | 0 | 0.1 | 0 | 0.1 | 0 | 0.5 | 0 | 0.3 | 0 | 0.1 | 0 | 0 |
| American Indian/Alaska Native | 0.6 | 0.2 | 1.0 | 0.2 | 0 | 0.2 | 2.4 | 0.2 | 2.3 | 0.2 | 1.8 | 0.2 | 0.2 |
| Other/unknown | 0.1 | 0.1 | 0.9 | 0.1 | 0 | 0.1 | 1.6 | 0.1 | 1.0 | 0.1 | 0.6 | 0.1 | 0.1 |

| | | | | | | | | | | | | | |
|---|---|---|---|---|---|---|---|---|---|---|---|---|---|
| **US census region, by state, %** | | | | | | | | | | | | | |
| Northeast | 0.8 | 0.1 | 1.6 | 0.1 | 0.8 | 0.1 | 2.5 | 0.1 | 2.4 | 0.1 | 1.6 | 0.1 | 0.1 |
| West | 0.5 | 0.1 | 0.9 | 0.1 | 0.4 | 0.1 | 1.5 | 0.1 | 1.5 | 0.1 | 1.0 | 0.1 | 0.1 |
| Midwest | 0.7 | 0.1 | 1.8 | 0.1 | 1.3 | 0.1 | 2.5 | 0.1 | 2.2 | 0.1 | 1.6 | 0.1 | 0.1 |
| South | 0.6 | 0.1 | 1.6 | 0.1 | 1.0 | 0.1 | 2.0 | 0.1 | 1.7 | 0.1 | 1.1 | 0.1 | 0.1 |
| **County vulnerability scores [c]** | | | | | | | | | | | | | |
| Highest vulnerability | 0.6 | 0.2 | 3.7 | 0.2 | 0.1 | 0.2 | 2.1 | 0.2 | 2.8 | 0.2 | 2.3 | 0.2 | 0.2 |
| Other | 0.6 | 0.1 | 1.4 | 0.1 | 0.7 | 0.1 | 2.1 | 0.1 | 1.8 | 0.1 | 1.3 | 0.1 | 0.1 |

Abbreviations: HBV, hepatitis B virus; HCV, hepatitis C virus, M, million. [a] Diagnosis coded as ≥1 inpatient or outpatient claim. [a] Defined as ≥1 diagnosis code in the inpatient claims or ≥2 outpatient claims spaced ≥1 month apart. [b] Defined as ≥1 diagnosis code in the inpatient or outpatient claim. [c] County vulnerability scores were reported by Van Handel et al in 2016. The top 5% of counties with the highest vulnerability scores were classified as *Highest vulnerability*, and the remaining counties were classified as *Other*.

**Table S4.** Adjusted Odds Ratios (aORs) and 95% Confidence Intervals (CIs) for Association of Opioid Overdose Claims with ≥1 HIV, HBV, or HCV Infection Among Medicare Beneficiaries with Non-overdose Opioid-related Claims, United States, 2015.

| **Characteristic** | **Opioid Overdose** | |
|---|---|---|
| | **aOR** | **95% CI** |
| **Age (years)** | | |
| ≤64 | 1.8 | 1.7–2.0 [a] |
| 65–74 | 1.5 | 1.4–1.7 [a] |
| ≥75 | Ref | Ref |
| **Sex** | | |
| Male | 1.0 | 0.9–1.0 [a] |
| Female | Ref | Ref |
| **Race/ethnicity** | | |
| Non-Hispanic white | Ref | Ref |
| Non-Hispanic black | 0.8 | 0.7–0.8 [a] |
| Hispanic | 0.9 | 0.8–1.0 [a] |
| Asian/Pacific Islander | 0.8 | 0.6–1.0 |
| American Indian/Alaska Native | 0.9 | 0.8–1.1 |
| Other/unknown | 1.1 | 0.9–1.2 |
| **US census region** | | |
| Northeast | Ref | Ref |
| Midwest | 1.2 | 1.1–1.2 [a] |
| South | 0.8 | 0.7–0.8 [a] |
| West | 0.9 | 0.9–1.0 [a] |
| **County vulnerability score [b]** | | |
| Highest | 1.4 | 1.3–1.5 [a] |
| Other | Ref | Ref |
| **Infection** | | |
| **Any of these 5 infections** | | |
| ≥1 infection (primary predictor variable) | 2.0 | 1.9–2.1 [a] |
| None of the 5 infections | Ref | Ref |

Abbreviations: HBV, hepatitis B virus; HCV, hepatitis C virus, Ref, reference. [a] Significant for chi-square test at $p < 0.05$. [b] County vulnerability scores were reported by Van Handel et al in 2016. The top 5% of counties with the highest vulnerability scores were classified as *Highest vulnerability*, and the remaining 95% of counties were classified as *Other*.

**Table S5.** Adjusted Odds Ratios (ORs) and 95% Confidence Intervals (CIs) for Association of Heroin Overdose Claims Among Medicare Beneficiaries With Non-overdose Opioid-related Claims, United States, 2015.

| Characteristic | Heroin Overdose | |
|---|---|---|
| | **aOR** | **95% CI** |
| **Age (years)** | | |
| ≤64 | 8.5 | 6.2–11.8 [a] |
| 65–74 | 3.4 | 2.4–4.7 [a] |
| ≥75 | Ref | Ref |
| **Sex** | | |
| Male | 1.7 | 1.6–1.8 [a] |
| Female | Ref | Ref |
| **Race/ethnicity** | | |
| Non-Hispanic white | Ref | Ref |
| Non-Hispanic black | 1.3 | 1.2–1.4 [a] |
| Hispanic | 1.4 | 1.2–1.6 [a] |
| Asian/Pacific Islander | 0.6 | 0.3–1.2 |
| American Indian/Alaska Native | 0.8 | 0.5–1.2 |
| Other/unknown | 1.3 | 1.0–1.7 [a] |
| **US census region** | | |
| Northeast | Ref | Ref |
| Midwest | 1.0 | 0.9–1.1 |
| South | 0.3 | 0.2–0.3 [a] |
| West | 0.4 | 0.3–0.4 [a] |
| **County vulnerability score [b]** | | |
| Highest vulnerability | 1.8 | 1.5–2.2 [a] |
| Other | Ref | Ref |
| **Infection (primary predictor variable)** | | |
| **HIV** | | |
| Yes | 0.9 | 0.8–1.1 |
| No | Ref | Ref |
| **Acute HBV** | | |
| Yes | 2.4 | 1.8–3.0 [a] |
| No | Ref | Ref |
| **Chronic HBV** | | |
| Yes | 1.4 | 1.0–2.0 [a] |
| No | Ref | Ref |
| **Acute HCV** | | |
| Yes | 1.5 | 1.2–1.8 [a] |
| No | Ref | Ref |
| **Chronic HCV** | | |
| Yes | 2.7 | 2.5–3.0 [a] |
| No | Ref | Ref |

Abbreviations: HBV, hepatitis B virus; HCV, hepatitis C virus. [a] Significant for chi-square test at $p < 0.05$. [b] County vulnerability scores were reported by Van Handel et al in 2016. The top 5% of counties with the highest vulnerability scores were classified as *Highest vulnerability*, and the remaining 95% of counties were classified as *Other*.
